# Humeral Shaft Fracture Sustained During Arm Wrestling with Review of Factors Contributing to its Causation

**DOI:** 10.5704/MOJ.2111.003

**Published:** 2021-11

**Authors:** KC Pande, NMH Nishat, SML Afzal, L Ishak

**Affiliations:** 1 Department of Orthopaedics, Raja Isteri Pengiran Anak Saleha Hospital, Bandar Seri Begawan, Brunei Darussalam; 2 PAPRSB Institute of Health Sciences, Universiti Brunei Darussalam, Brunei Darussalam; 3 Department of Orthopaedics, Suri Seri Begawan Hospital, Kuala Belait, Brunei Darussalam

**Keywords:** arm wrestling, humeral fracture, biomechanics, electromyography

## Abstract

**Introduction:**

Humeral shaft fractures are the most common injury sustained in arm wrestling, and its various biomechanical, anatomical, kinematic and electromyographic aspects have been studied and reported. We present a series of six cases of humeral shaft fractures in the arm wrestlers and a review of basic science studies to determine the factors contributing to their causation.

**Materials and methods:**

Six humeral shaft fractures associated with arm wrestling were treated between December 2018 and January 2020. The medical records and radiographs were retrospectively reviewed. In addition, the characteristics of the patients, their opponents, and the fractures were noted in a pre-designed data sheet.

**Results:**

There were six men with an average age of 27.5 years (SD ± 8.9). All were amateurs who were occasional arm wrestlers. Three fractures each were sustained in the sitting and standing position, four in the losing phase, one in the winning phase, and one in the stalling phase. The dominant side humerus was involved in all but one case. The fracture types were 12-A1 (n=4); 12-B1 (n=1); 12-A2 (n=1). Three fractures were treated by open reduction and internal fixation, while three were treated conservatively with satisfactory healing.

**Conclusion:**

Humeral shaft fractures in arm wrestling are common in amateurs. There is no association of the fracture with the position of the players or the phase of the match. However, arm wrestlers should be aware of this complication and should receive proper guidance to reduce the risk of humeral shaft fractures.

## Introduction

Arm wrestling is a common sport across the world. Its popularity stems from the fact that it is thrilling and no complicated equipment is needed^1^. Two competitors face each other, either sitting or standing, with their hands gripped. Then, with the elbows on the table, each tries to force the opponent's arm down to the table. Unfortunately, humeral shaft fractures and other injuries can occur during a competition^2^.

In Brunei Darussalam, arm wrestling is called *berudi* or *berambit* in Brunei Malay or *Gusti Lengan* in standard Malay. The official arm wrestling association in the country was formed recently, and a championship was organised with arm wrestlers from Brunei, Indonesia and the East Malaysian states of Sabah and Sarawak, confirming its popularity in South-East Asia^3^.

We report a series of humeral shaft fractures in arm wrestlers in Brunei Darussalam and explore the factors contributing to the fracture.

## Materials and Methods

A total of six cases presenting between December 2018 and January 2020, with humerus fracture sustained during arm wrestling, were identified. The medical records and radiographs were retrospectively reviewed. The follow-up ranged from 3 to 17 months. The AOOTA classification was used to classify the fractures^4^.

Additional information about the patients and their opponents related to the fracture was noted in a pre-designed datasheet. This was done through a telephone interview where the patients were informed of the purpose of the study and gave informed consent for the inclusion of their details in the study.

These patients with their humeral fractures treated surgically or conservatively, were compared for time when healing was noticed and when mobilisation could be started. The range of movements and the return to gym training were recorded at the last follow-up. Residual deformity at the fracture site was assessed on the final follow-up radiograph.

## Results

The characteristics of the subjects are presented in Table I. The average age and SD of the cases were 27.5years ± 8.9 (range 20.4 to 44.7). Five patients were under 30 years of age.

**Table I:** Subject, opponent and fracture characteristics of the cases

Case				Subject				Opponent			Fracture		
	Age	A/P	Freq.	Training	Steroid	Game	Position	A/P	Strength	Dom.	Side	Type	Mgt.
1	29.5	A	Occ	Yes	No	2	Standing	A	Same	R	R	12-A2	Sx
2	22.11	A	Occ	Yes	No	2	Sitting	A	Same	L	L	12-A1	Sx
3	20.4	A	Occ	Yes	No	7	Standing	A	Same	R	R	12-A1	Con.
4	24.3	A	Occ	No	No	1	Sitting	A	Same	R	R	12-A1	Sx
5	44.7	A	First	No	Yes	1	Sitting	P	Stronger	R	R	12-B1	Con.
6	24.5	A	First	No	No	1	Standing	A	Same	R	L	12-A1	Con.

Abbreviations: Age - Years, A/P - Amateur / Professional, Freq - Frequency of play, Occ - Occasional, Game - Number of game when fracture was sustained, Dom - Dominant extremity, R - Right, L - Left, Mgt. - Management, Sx - Surgical, Con. - Conservative

All the cases were amateurs, with four reporting involvement in arm wrestling occasionally and two taking part for the first time. Three reported undergoing some strength training in the gymnasium, while three had not undergone any training. Only one patient reported warming up before the match (Case 2). Three cases sustained the fracture of the humerus during their first game (Case 5,6,7; Case 5, 6 being first-time players), two during their second game they had participated in (Case 1,2; Case 2 taking part in a winner stay competition), and one patient sustained the fracture during the 7th game when he was sparring (Case 3). The fractures occurred in three each in the sitting and standing position, four when they were losing the match, one in the winning phase, and one during stalling.

In five cases, the opponent was also an amateur player, while in one case, the match was against a professional player (Case 5). Finally, in four cases, the opponent was deemed to have similar strength and, in two cases, stronger than the patient.

**Fig 1:**
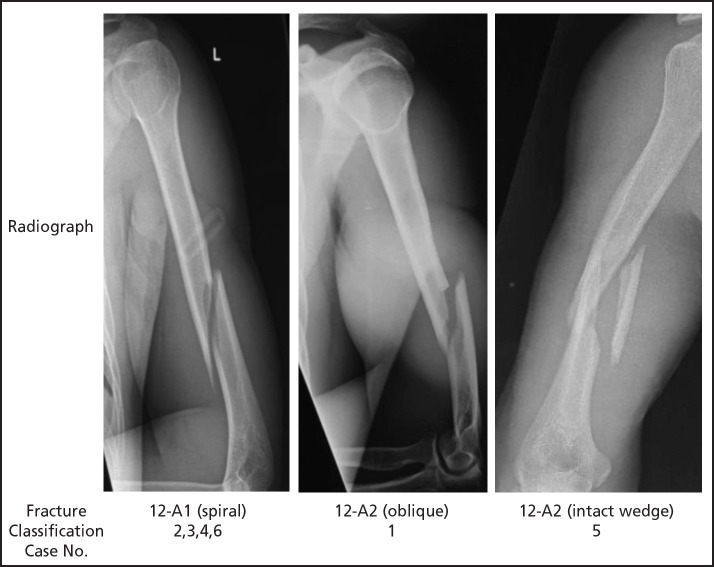
Pattern of fracture according to AOOTA Classification.

The dominant side was fractured in five cases, while in one, the less dominant side was involved. Of the fracture pattern, there were four 12-A1 (spiral), one 12-A2 (oblique) and one 12-B1 (intact wedge) (Fig. 1). None of these had associated radial nerve palsy.

After discussing the treatment options, three patients opted to have open reduction and internal fixation while the remaining three were treated conservatively. All fractures healed in optimal time.

Evidence of healing of the fracture on radiographs was seen between two to three months in the subjects treated by internal fixation and in those treated conservatively. In subjects treated by internal fixation, mobilisation could be started at one month post-surgery, compared to more than two months in those treated conservatively.

Subjects treated by internal fixation had no residual deformity. They regained full range of movements, while all the cases treated conservatively had residual varus deformity at the fracture site. In two subjects, there was a limitation of rotational movements at the shoulder at the last follow-up. At the last follow-up, only one subject treated by internal fixation had resumed gym training. None of the six subjects expressed a desire to return to arm wrestling.

## Discussion

Fracture of the shaft of the humerus is the most commonly reported injury in arm wrestling. Several case series^1,5-12^ and individual case reports are available in the literature^13,14^.

The other injuries of the upper limb associated with arm wrestling included fractures of the medial epicondyle^15,16^, radial neck^17^, radial shaft^18^, and the olecranon^19^ and the rupture of the subscapularis^20^.

Humeral shaft fractures in arm wrestling are mostly reported in men as in this series, while some are in female arm wrestlers^7^. The age distribution of our cases is similar to that reported in the literature^1,2,7,9-11^.

It is suggested that amateurs are more prone to get humeral fractures as they use an incorrect technique of stabilisation of the arm at the glenohumeral joint^9^. This may be a contributory factor in the causation of humeral fracture in our series, where all six subjects were amateur arm wrestlers. Though the humeral fracture in arm wrestling has been reported to occur during different phases of the match, as in this study, Ogawa and Ui have noted that the consistent factor in the causation has been a competitor applying full force to decide a match. In the present series, four of the six cases sustained a fracture when the opponents were applying maximal force as they were losing the match^7^.

The strength of the opponent does not influence the occurrence of humeral shaft fracture^1,7^. In the present series, only in one case was the opponent deemed to be stronger as he was a professional arm wrestler, while in the other cases, the opponents were considered to have similar built and strength. This is in contrast to a kinematic and electromyographic study which suggested that the strength of the pectoralis major muscle might offer a participant a winning advantage in the competition^21^.

An equal number of fractures were sustained in the sitting and standing position in our study. No effect of the position taken during the arm wrestling match was noted. Nevertheless, it was proposed that a position that allowed shifting of the centre of gravity to apply a higher force might contribute to the fracture^7^.

The pattern of fracture most often seen is a spiral fracture of the distal humerus (12-A1), similar to the present study. We had one case of a fracture of the humerus with a large butterfly fragment (12-B1), which is the second most common pattern reported^2^. Correira *et al* (2018) identified two clear patterns of injuries depending on age. In adolescents under 18 years, a fracture of the medial epicondyle associated with an ulnar nerve palsy in some, is common while in adults, a spiral fracture of the distal third of the humerus is most common^2^. Ogawa and Ui proposed that this is due to tension on the common flexor origin on the unfused and weak growth plate of the distal humerus in adolescents, which fails before the force is transmitted to the shaft of the distal humerus^7^.

In some cases, the humerus fracture is complicated by radial nerve palsy with an incidence between 1.8 to 20%^1,2,7,9^. None of the cases in our study was complicated by radial nerve palsy.

Mayfield and Egol compared fractures of the humerus sustained during arm wrestling with those from other mechanisms that were treated non-operatively. Except for earlier healing of fracture in the arm wrestlers, no differences were found^12^. In some series, the patients were treated surgically^9^. Some non-surgical treatment was carried out^12^ while others have included patients treated by both modalities^7,10^, all reporting satisfactory outcomes. A study comparing the outcome of surgical and non-operative management of humeral fractures sustained during arm wrestling reported similar results^10^. Three cases each in the present series were treated non-surgically and by open reduction and internal fixation with adequate healing.

A large amount of torsional force is generated in the arm during an arm-wrestling match due to the active internal rotation of the arm against the opponent with the elbow fixed in flexion^7^. Moon *et al*^5^ and Whitaker^22^ opined that the humeral fracture results from a combination of bending movement, axial compression and torsional forces.

Ogawa and Ui have proposed that under dominant force during a match, the internal rotators of the opponent are under stress and change from concentric contraction to eccentric contraction-relaxation resulting in increased rotational torque and subsequent fracture^7^.

In a study of tennis players, pronounced hypertrophy of the humerus has been reported on the playing side, confirming that the strength of the humerus is related to the muscular forces acting on it^23^. The humeral shaft fracture is known to occur in the weaker non-dominant side, as was seen in one of our cases. This would suggest that the occurrence of fracture does not depend only on muscle strength^7^. Competitors with a longer forearm may have a moment-torque advantage with the elbow flexed^8^.

Kruczynski *et al* conducted a biomechanical analysis using finite element analysis (FEM) based on computer tomography scans of the humerus. During simulated arm wrestling, the maximum bone stress from torsional loading was 60 MPa and was located 115mm above the elbow on the posteromedial aspect of the humerus. The forces of acting muscles were noted to cause significant loading in the distal one-third of the humerus. These two factors result in the typical spiral fracture of the humerus in the distal one-third of humerus commonly seen in arm wrestling^9^. Pedrazzini *et al* used the strength of the material concept and computerised tomography and bone density scan to study the humerus and showed that the bone mineral content and ratio of outer to inner diameter is less in the distal humerus than rest of the humerus^24^.

Marks *et al* conducted a bone morphology study using axial and longitudinal cuts. It was noted that there is a change in the shape of the cross-section of the humerus from a tubular one to a triangular one in the middle-distal third. Spiral orientation of the bone morphological structures was observed on the longitudinal section in the same area. Additionally, in the experimental model, they observed a spiral fracture by applying pure internal rotational force mimicking a real-life situation in the distal shaft of the humerus. The authors concluded that part of the shaft of the humerus where its cross-section changes act as an area of least resistance, and the spiral structure of the bone favours the occurrence of a spiral fracture on the application of a rotational load during arm wrestling^25^.

During arm wrestling, the deltoid, biceps brachii and brachialis maintain the arm flexion while the pectoralis major and subscapularis act as internal rotators^9^. An electromyographic study in simulated conditions confirmed that pectoralis major and flexor carpi ulnaris are the agonists while biceps brachii and pronator teres play a secondary role. It was also seen that the electrical activity depended on the load and the position of the upper limb^26^. The importance of the pectoralis major was also confirmed in another kinematic and electromyographic analysis where it showed a higher muscle activity in the winning position than in the losing position^21^. A study comparing the shoulder internal rotation strength in arm wrestlers confirmed that the mean peak torque values for internal rotators of the shoulder were higher in winners than losers^27^. Other muscles which have been shown to allow the winner to gain an advantageous position include the flexor carpi ulnaris, with its role in wrist flexion, and the strength of biceps and brachialis, with their role in elbow flexion^21,27^.

In addition to these factors, other factors that can lead to the humerus fracture include poor posture, inadequate training, hypertrophy of muscles and inefficient motor control^24^.

Napp *et al* have reported a humerus shaft fracture in an arm wrestler with a history of anabolic steroid use, where an imbalance between the strength of the muscles and the thickness of the humeral cortex caused by anabolic steroid use may predispose such patients to the fracture^28^. In the present series, only one patient reported using anabolic steroids, and he sustained the fracture while taking part for the first time against a professional arm wrestler.

Marks *et al* proposed some rules for an arm-wrestling match to reduce the chances of a humeral shaft fracture. These include a selection of competitors matched for height and weight, avoiding sudden ending of the match and a proper technique avoiding stabilisation of the arm at the glenohumeral joint^25^. There is evidence that when matches are played under competition rules, even by amateurs, the chance of sustaining a fracture is less^6^.

It is clear from these studies that typical humeral shaft fracture in arm wrestling results from a combination of various factors. The two most widely studied being the typical anatomical and material properties of the distal humerus and the force generated by the action of the various muscles, mainly the internal rotators of the shoulder.

## Conclusion

Fractures of the humeral shaft seen in arm wrestlers are common in amateurs, and the participants should be made aware of this risk. These result from a rotation torque, axial loading and bending. The typical pattern of fracture noted is due to the anatomical, and material characteristics of the humerus at the middle and distal one-third and the tension exerted when the internal rotators change from concentric to eccentric contraction. Internal fixation of fracture allows for early mobilisation, avoids residual deformity and allows a full return of movements, though this may not determine an ability to return to arm wrestling. Information from basic science studies should be used to frame the match rules and train the arm wrestlers to prevent these fractures.
